# Time for Precision: A World Without Susceptibility Breakpoints

**DOI:** 10.1093/ofid/ofy282

**Published:** 2018-10-31

**Authors:** Justin C Bader, Elizabeth A Lakota, David R Andes, Christopher M Rubino, Paul G Ambrose, Sujata M Bhavnani

**Affiliations:** 1 Institute for Clinical Pharmacodynamics, Schenectady, New York; 2 University of Wisconsin, Madison, Wisconsin

**Keywords:** in vitro susceptibility testing criteria, pharmacokinetic, pharmacokinetics-pharmacodynamics, precision medicine, susceptibility breakpoints

## Abstract

Interpretive criteria for in vitro susceptibility testing criteria, “susceptibility breakpoints,” underpin the evaluation and selection of antimicrobial regimens. However, despite their strengths, susceptibility breakpoints are a relatively blunt instrument employed to address an extremely complex question—what is the likelihood of treatment success for individual patients? With regard to evaluating patients on a case-by-case basis, breakpoints merely allow us to account for pathogen susceptibility. This approach precludes consideration of drug exposures achieved in patients, thus overlooking half of the equation for predicting treatment success. Herein, we propose the framework for considering both pathogen- and patient-specific information to provide clinicians a means of evaluating antimicrobial regimens for individual patients through tools automating pharmacokinetic-pharmacodynamic target attainment analyses. Implementing these tools along with their acceptance by professional organizations will allow for a shift in the paradigm for how antimicrobials are selected and dosed—toward patient-centered care through precision medicine.

## THE WAY OF THE WORLD

We live in a world awash with antimicrobial resistance. As the prevalence rates of multidrug-resistant, extensively drug-resistant, and pandrug-resistant pathogens have increased, clinicians have needed to consider new antimicrobials or administer higher doses of standard agents to ensure adequate efficacy as once-efficacious therapies have fallen to the wayside. Antimicrobial stewardship efforts tasked with combating these “super bugs” are underpinned by in vitro susceptibility testing criteria (ie, susceptibility breakpoints), which have served as a surrogate for predicting the likelihood of antimicrobial treatment success for over half a century. However, despite their strengths, susceptibility breakpoints are a relatively blunt instrument employed to address an extremely complex question. In principle, susceptibility breakpoints are fixed minimum inhibitory concentration (MIC) values or zones of inhibition at which an antimicrobial agent, based on approved regimens, is considered to have a high, moderate, or low likelihood of success (susceptible, intermediate, and resistant, respectively) in treating a pathogen of interest.

For evaluating patients on a case-by-case basis, susceptibility breakpoints allow us to account for pathogen susceptibility but preclude the consideration of drug exposures on the patient level. Most often, clinicians must use dose as a surrogate for drug exposures given that they do not have access to pharmacometric technologies at the point of care. Consequently, a great need exists for tools capable of estimating drug exposures and integrating them with pathogen susceptibility information (ie, MICs) to better predict treatment success in individual patients.

Importantly, a shift away from the “one size fits all” approach for regimen selection is already underway across various fields of medicine. In its place, precision medicine, the practice of accounting for individual and disease state variability to optimize treatment decisions, is being adopted. This transition is most rapidly occurring within the field of oncology, in which treatment courses are frequently selected based on the genomics of a patient’s afflicting cancer. In fact, large clinical trials have been initiated to evaluate the effectiveness of treatments selected on the basis of tumors’ molecular signatures rather than their organ site of origin [1].

Herein, we propose a framework for applying the principles of precision medicine to the treatment of infectious diseases, wherein clinicians could be provided a means of evaluating antimicrobial regimens on the basis of more than just pathogen susceptibility.

## THE CRACKS IN THE ROAD

**Figure 1.**
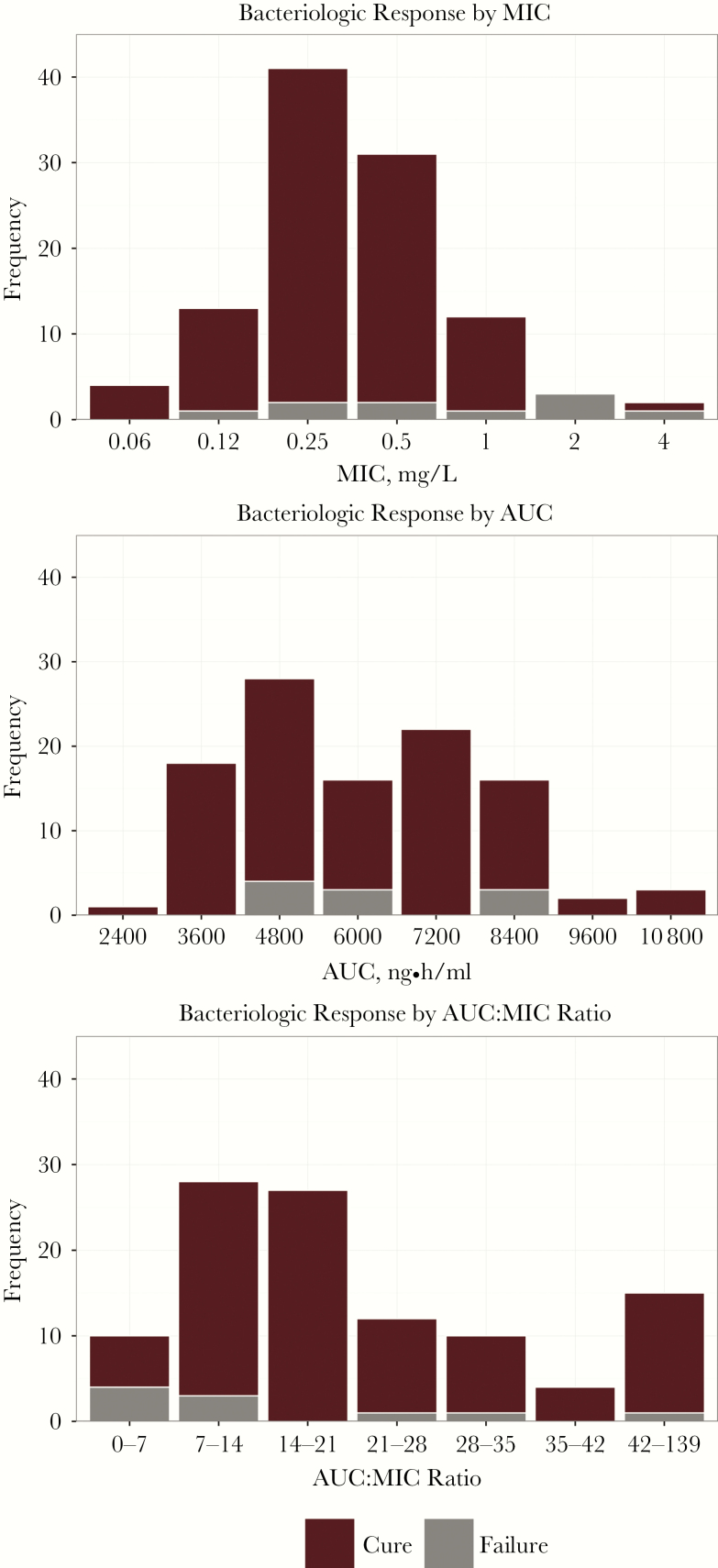
Distributions of bacteriologic response by minimum inhibitory concentration (MIC), area under the concentration-time curve (AUC), and AUC:MIC ratio values for 106 isolates obtained from 71 tigecycline-treated patients with complicated intra-abdominal infections.

Although some data demonstrate the relationship between increased MIC and reduced efficacy of antimicrobial agents [2–4], such trends have not been observed in other reports [5–7]. This conflicting evidence may indicate that pathogen susceptibility alone is not an optimal predictor of treatment outcomes. The basis for this premise is that only half of the equation for predicting treatment success is considered when MIC is evaluated alone. A prior evaluation of clinical trial data for tigecycline-treated patients with complicated intra-abdominal infections supports this hypothesis [8]. Figure 1 shows bacteriologic response by MIC of the baseline pathogen, ranges of individual patient exposures as represented by steady-state area under the concentration-time curve (AUC) values, and the integration of these elements as conveyed by ratios of AUC-to-MIC values (AUC:MIC ratio). When evaluating bacteriologic response by MIC or AUC alone, no trend was apparent. However, when evaluated by AUC:MIC ratio, a clear trend—greater frequency of failures among patients with lower AUC:MIC ratios—was evident, thus demonstrating that drug exposure indexed to drug potency (MIC) is more informative than either variable alone.

Organizations tasked with establishing susceptibility breakpoints (eg, the Clinical and Laboratory Standards Institute [CLSI]) have recognized the importance of accounting for drug exposures in patients when establishing susceptibility breakpoints. Over the past 2 decades, great strides have been made to this effect, with the increased use of population pharmacokinetic (PK) models, preclinical and clinical pharmacokinetic-pharmacodynamic (PK-PD) models for efficacy, and Monte Carlo simulation to inform breakpoint decisions. Moreover, increased focus is now being given to the influence of drug exposure at the effect site (eg, epithelial lining fluid concentrations to evaluate treatment regimens for patients with respiratory infections), the associated bacterial burden, and/or the severity of infection when considering PK and/or PK-PD inputs for analyses to support susceptibility breakpoint recommendations. However, the trouble with relying upon susceptibility breakpoints for clinical decisions stems from how these interpretative criteria are defined.

CLSI describes a susceptible breakpoint as an MIC at which “isolates are inhibited by the usually achievable concentrations of an antimicrobial agent when the dosage recommended to treat the site of infection is used” [9]. And herein lies the crucial limitation within the current paradigm. Susceptibility breakpoints are a relatively static metric for predicting treatment success. These end points evaluate pathogen susceptibility on the basis of fixed exposures for a given regimen or set of regimens as observed in a typical patient population. Accordingly, they are limited in their ability to individualize treatment decisions (eg, dose, duration, and frequency) on the basis of patient-specific factors.

## EMBRACING SOME NOT SO NEW TOOLS FOR ANTIMICROBIAL THERAPY SELECTION

Fortunately, the answer to overcoming the limitations of susceptibility breakpoints is less of an unknown than one might suspect—in fact, the necessary tools are already in use and are widely discussed in the literature. The approach needed integrates population PK models, exposure-response relationships for efficacy, and pathogen susceptibility data through Monte Carlo simulation. This allows for the assessment of percent probabilities of attaining drug exposures indexed to MIC, which are associated with efficacy (PK-PD indices) relative to a simulated patient population administered a given antimicrobial regimen of interest. By leveraging this approach, commonly known as PK-PD target attainment analyses, we can predict the likelihood of achieving positive therapeutic outcomes in a seemingly infinite number of scenarios. But how does this work?

Today, nearly all drug developers collect data from subjects and patients enrolled in clinical studies to construct population PK models. These models characterize the disposition of drugs in plasma and other exposure matrices and can be used to identify significant patient covariates and quantify their magnitudes of effect on PK. Through the use of a population PK model and Monte Carlo simulation, concentration-time profiles for a given drug can be generated for a simulated population of patients, whether this may be typical patients or a specific population of interest (eg, patients with renal impairment). These simulated profiles, in conjunction with pathogen susceptibility data, can be used to generate PK-PD indices for efficacy (eg, time that antimicrobial concentrations are above the MIC [%T>MIC], the ratio of the peak concentration to the MIC [C_max_:MIC ratio], or the AUC:MIC ratio) for each patient. Ultimately, a calculation of the proportion of simulated patients achieving the PK-PD target of interest at a given MIC yields the probability of PK-PD target attainment.

The value of this approach will only become more apparent over time as rapid diagnostic technologies are further developed and implemented. In such situations, the pathogen of interest will be identified early in therapy, before susceptibility information is available. This is beneficial given that PK-PD target attainment analyses can be performed without measured MICs. Through integration of local or national in vitro surveillance data, probabilities of PK-PD target attainment weighted over MIC distributions can be determined, thus enabling clinicians to better evaluate and select empiric therapy.

## IS IT TIME TO SELECT THERAPY SPECIFIC TO THE PATIENT?

Outside of antimicrobial stewardship programs, it is far too often that clinicians review susceptibility panels and dismiss antimicrobials with “intermediate” or “resistant” susceptibilities. However, PK-PD target attainment analyses allow for more informed assessments of such scenarios.

**Figure 2.**
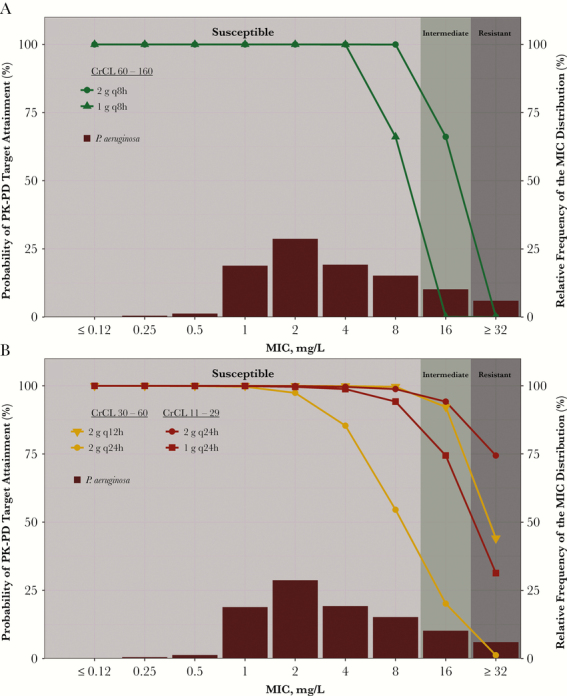
Percent probabilities of pharmacokinetic-pharmacodynamic target attainment by minimum inhibitory concentration (MIC) on day 1 based on a free-drug plasma %T>MIC ratio target associated with a 1-log_10_ colony-forming unit reduction from baseline for *Pseudomonas aeruginosa* among simulated patients with normal renal function (A) and moderate to very severe renal impairment (B) by cefepime regimen, overlaid over a North American *P. aeruginosa* MIC distribution (n = 1639, SENTRY Program, 2005–2007).

Using cefepime day 1 free-drug plasma %T>MIC values and a free-drug plasma %T>MIC target associated with a 1-log_10_ colony-forming unit reduction from baseline for *Pseudomonas aeruginosa*, Figure 2, A and B, depicts the probabilities of PK-PD target attainment by MIC among simulated patients with normal renal function and moderate to severe renal impairment, respectively, administered regimens adjusted according to renal function [10, 11]. Figure 2A demonstrates that the probability of achieving therapeutic exposures can vary greatly across regimens even in a population with normal renal function, as evidenced by the probabilities of PK-PD target attainment for the 1- and 2-g q8h regimens at an MIC of 8 mg/L (66.1 and 100%, respectively), which is defined as susceptible [9]. Figure 2B, on the other hand, illustrates another important principle. By accounting for renal impairment, high probabilities of PK-PD target attainment can be achieved even for isolates considered intermediate-resistant. Probabilities of PK-PD target attainment at the intermediate-resistant MIC for patients with moderate and severe renal impairment were as high as 92.2% and 94.2%, respectively.

Instead of assuming that an antimicrobial agent is effective simply because a clinical isolate has been categorized as “susceptible” or that it does not warrant use because it is considered “intermediate-resistant,” results of PK-PD target attainment analyses enable us to better delineate what the antimicrobial agent in question has to offer.

## TAKING THE APPROACH ONE STEP FURTHER

As previously argued by Roberts et al., the approach described above is of greatest importance for the treatment of critically ill patients, for whom PK variability is greatest and outcome is dependent on optimized antimicrobial therapy [12]. For such patients, interest in utilizing therapeutic drug monitoring (TDM), which provides the opportunity to better predict exposures, is growing. Specifically, Bayesian-based adaptive feedback control algorithms can utilize relevant demographic information and drug concentrations obtained through TDM (even as few as 1 sample) to estimate individual-specific PK parameters for use in subsequent simulations. Of note, although Bayesian analyses are a useful extension of the techniques described herein, it is unknown whether the effort and expense required to obtain their prerequisite data (ie, collecting and assaying patient drug concentrations) outweigh the benefits gained for all patients, or if their use should be limited to the critically ill and those receiving agents with narrow therapeutic indices.

Moreover, PK-PD target attainment analyses can be further leveraged through the use of targets for safety, when available. These data, in conjunction with targets for efficacy, could be used to optimize regimen selections based on therapeutic windows, most notably for antimicrobials with narrow therapeutic indices or those with substantial drug-related adverse events.

## BRINGING THE THEORY TO THE CLINIC

To date, utilizing PK-PD target attainment analyses in a clinical setting has been challenging. The complexity of the mathematics and programming involved and the technology needed to perform simulations in the clinic have hindered clinicians’ access. Moreover, collecting the prerequisite information needed to conduct these analyses is a time-consuming chore. However, in light of the major technologic advances made over the past decade, we believe adopting a PK-PD-driven approach to antimicrobial regimen selection is now feasible. Microprocessors capable of automating tens of thousands of simulation runs per second are nearly ubiquitous in today’s world, making what once would have been considered a pipe dream into reality. Likewise, the capacity and sophistication of electronic health record systems have increased many fold over this period.

**Figure 3.**
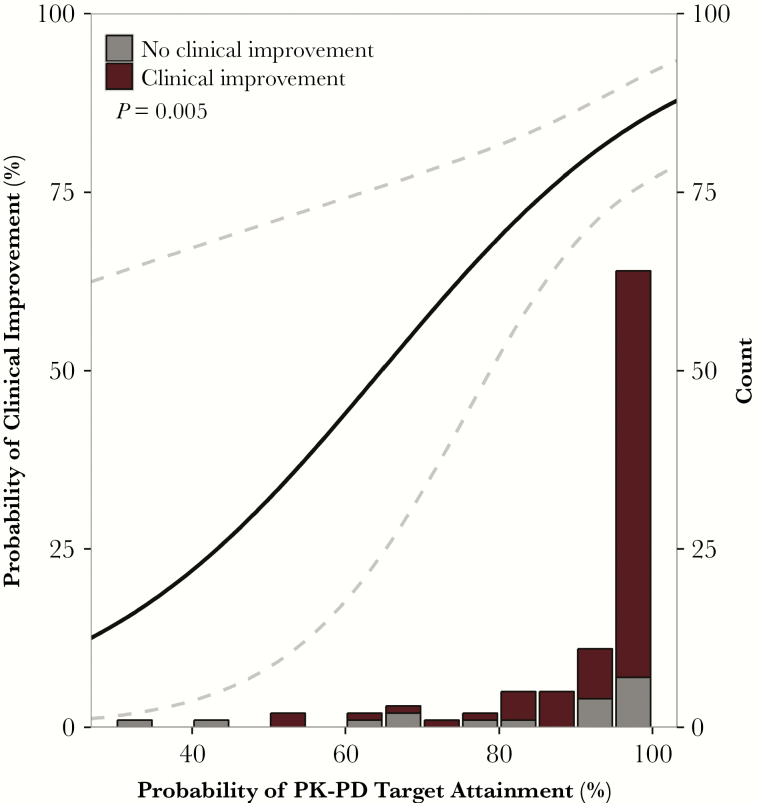
Logistic regression analysis evaluating the relationship between the probability of pharmacokinetic-pharmacodynamic target attainment and probability of clinical improvement 48 hours after therapy selection. Abbreviations: PK-PD, pharmacokinetic-pharmacodynamic.

In fact, prior evaluations of data collected from a computerized decision support system that performed PK-PD target attainment analyses demonstrated the utility of this approach. Analyses were performed to characterize the relationship between the probability of PK-PD target attainment for a patient’s prescribed treatment regimen relative to clinical outcomes at follow-up 48 hours after therapy selection [13]. When these data were fit using logistic regression, a significant relationship was identified between the probabilities of PK-PD target attainment and clinical improvement (Figure 3). For every 10% increase in PK-PD target attainment achieved, clinical improvement was predicted to be 1.63 times more likely. Ultimately, the predicted probability of clinical improvement at 48 hours for a regimen with a 90% probability of PK-PD target attainment was 81.3%.

At the bedside, this information could provide more precise estimates and informed treatment options for physicians and antimicrobial stewardship teams. Given the demonstrated importance of both PK variability in patients and time to effective therapy, PK-PD-optimized drug and dosing regimen decisions are likely to have the greatest impact on patient outcome relative to other antimicrobial stewardship interventions.

## THE HURDLES AHEAD AND WHERE WE GO FROM HERE

As stated at the onset, susceptibility breakpoints have been in use for over half a century and are central to the selection of antimicrobial regimens. Laboratory services for health care systems are structured around the use of susceptibility breakpoints. However, current standards for MIC testing pose some challenges to implementing the above-described approach. For instance, data generated from automated antimicrobial susceptibility testing systems primarily utilize commercially prepared test panels, cards, or trays containing select antimicrobial agents evaluated across prespecified MIC dilutions. These test panels have limited capacity (~≤96 wells), thus limiting the number of agents and MIC dilutions that can be evaluated. To avoid incurring the substantial costs associated with purchasing additional testing equipment and panels, many manufacturers and health care institutions have opted to use limited panels of agents for testing over a select number of dilutions (typically corresponding to the susceptibility breakpoints or observed range of MICs for each agent) to “optimize” the data derived from each panel.

To truly leverage the above-described approach and provide probabilities of PK-PD target attainment for a given antimicrobial regimen, an accurate estimation of the “true” MIC is needed. However, in addition to the limitations discussed above, it is important to acknowledge that the MIC—the lowest concentration at which *observable* growth is inhibited—is a measure of convenience. This end point lacks a well-defined pathophysiologic basis, is measured in broad 2-fold dilutions, and is associated with great variability depending upon the testing conditions utilized for its measurement [14]. However, much of infectious diseases treatment is based upon evaluation of this imperfect but useful measure, which underscores the importance of sound judgment and clinical experience when selecting antimicrobial regimens, regardless of the tool used to predict the likelihood of treatment success. Consequently, progress made in the near term will require the use of MICs until a broader paradigm shift toward the use of a more precise and informative measure of drug potency that is appropriate for use at the point of care is attainable.

In summary, this paper has endeavored to demonstrate that the use of PK and PK-PD can provide the opportunity to extend the principles of precision medicine to the treatment of infectious diseases. Despite the oftentimes limited and imperfect data available to inform analyses, PK-PD target attainment analyses present a significant step toward a patient-centered approach to antimicrobial regimen selection and in turn, offer an evident advantage over traditional susceptibility breakpoints.

The key to success for the paradigm proposed is 3-fold. The first criterion will be making the approach described accessible. Fortunately, as others have described [15, 16], decision support systems integrating PK and PK-PD are already being integrated with electronic health record systems. The second criterion will be acceptance of this paradigm by the professional organizations responsible for drafting antimicrobial treatment and in vitro susceptibility testing criteria guidelines. We encourage these organizations to gradually refocus their efforts toward evaluating the inputs utilized for the PK- and PK-PD-based tools described herein. Fulfillment of these criteria, in addition to the third criterion, adoption by the general medical community, would pave the way for a new standard of practice to improve patient care.
